# Patients with stage 3 compared to stage 4 liver fibrosis have lower frequency of and longer time to liver disease complications

**DOI:** 10.1371/journal.pone.0197117

**Published:** 2018-05-10

**Authors:** Page Axley, Sandhya Mudumbi, Shabnam Sarker, Yong-Fang Kuo, Ashwani Singal

**Affiliations:** 1 Department of Internal Medicine, University of Alabama at Birmingham, Birmingham, Alabama, United States of America; 2 Department of Biostatistics, University of Texas Medical Branch, Galveston, TX, United States of America; 3 Division of Gastroenterology and Hepatology, University of Alabama at Birmingham, Birmingham, Alabama, United States of America; Harvard Medical School, UNITED STATES

## Abstract

**Background and aims:**

Advanced liver fibrosis is an important predictor of liver disease progression and mortality, and current guidelines recommend screening for complications of cirrhosis once patients develop F3 fibrosis. Our study compared liver disease progression and survival in patients with stage 3 (F3) and stage 4 (F4) fibrosis on liver biopsy.

**Methods:**

Retrospective study of patients with F3 or F4 on liver biopsy followed for development of liver disease complications (variceal bleeding, ascites, and hepatic encephalopathy); hepatocellular carcinoma, and survival (overall and transplant free survival).

**Results:**

Of 2488 patients receiving liver biopsy between 01/02 and 12/12, a total of 294 (171 F3) were analyzed. Over a median follow up period of 3 years, patients with F4 (mean age 53 years, 63% male) compared to F3 (mean age 49 years, 43% male) had higher five year cumulative probability of any decompensation (38% vs. 14%, p<0.0001), including variceal bleed (10% vs. 4%, p = 0.014), ascites (21% vs. 9%, p = 0.0014), and hepatic encephalopathy (14% vs. 5%, p = 0.003). F4 patients also had lower overall 5-year survival (80% vs. 93%, p = 0.003) and transplant free survival (80% vs. 93%, p = 0.002). Probability of hepatocellular carcinoma in 5 years after biopsy was similar between F3 and F4 (1.2% vs. 2%, p = 0.54).

**Conclusions:**

Compared to F4 stage, patients with F3 fibrosis have decreased risk for development of liver disease complications and better survival. Prospective well designed studies are suggested with large sample size and overcoming the limitations identified in this study, to confirm and validate these findings, as basis for modifying guidelines and recommendations on follow up of patients with advanced fibrosis and stage 3 liver fibrosis.

## Introduction

Chronic liver disease progresses through varying stages of fibrosis to cirrhosis.[1] The accepted “gold standard” for diagnosis of cirrhosis is liver biopsy. The METAVIR scoring system developed in France in 1993, has been adapted for histological staging of liver disease in most etiologies of chronic liver disease.[2–5] According to this staging system, stage 3 fibrosis (F3) is defined as “bridging fibrosis” evidenced by fibrotic bridging that extends across lobules, between portal areas, and between portal areas and central veins. It is an extension from stage 2 fibrosis in which fibrosis is limited to periportal or perivenular areas. The hepatic architecture remains relatively intact. When fibrosis progresses to and distorts the liver architecture with formation of nodules, it is considered stage 4 fibrosis (F4) or cirrhosis.[6]

In clinical practice, patients with F3 are approached similar to patients with compensated cirrhosis regarding screening for hepatocellular carcinoma (HCC) and for varices.[7] Data are limited on the liver disease progression comparing F3 and F4 stages. We performed this study with primary aim to compare F3 patients with F4 confirmed on liver biopsy for time to development of liver-disease related complications such as variceal bleeding, hepatic encephalopathy, ascites, and HCC. We also compared the two groups for overall and transplant free survival.

## Methods

### Study population

**Fig 1:**
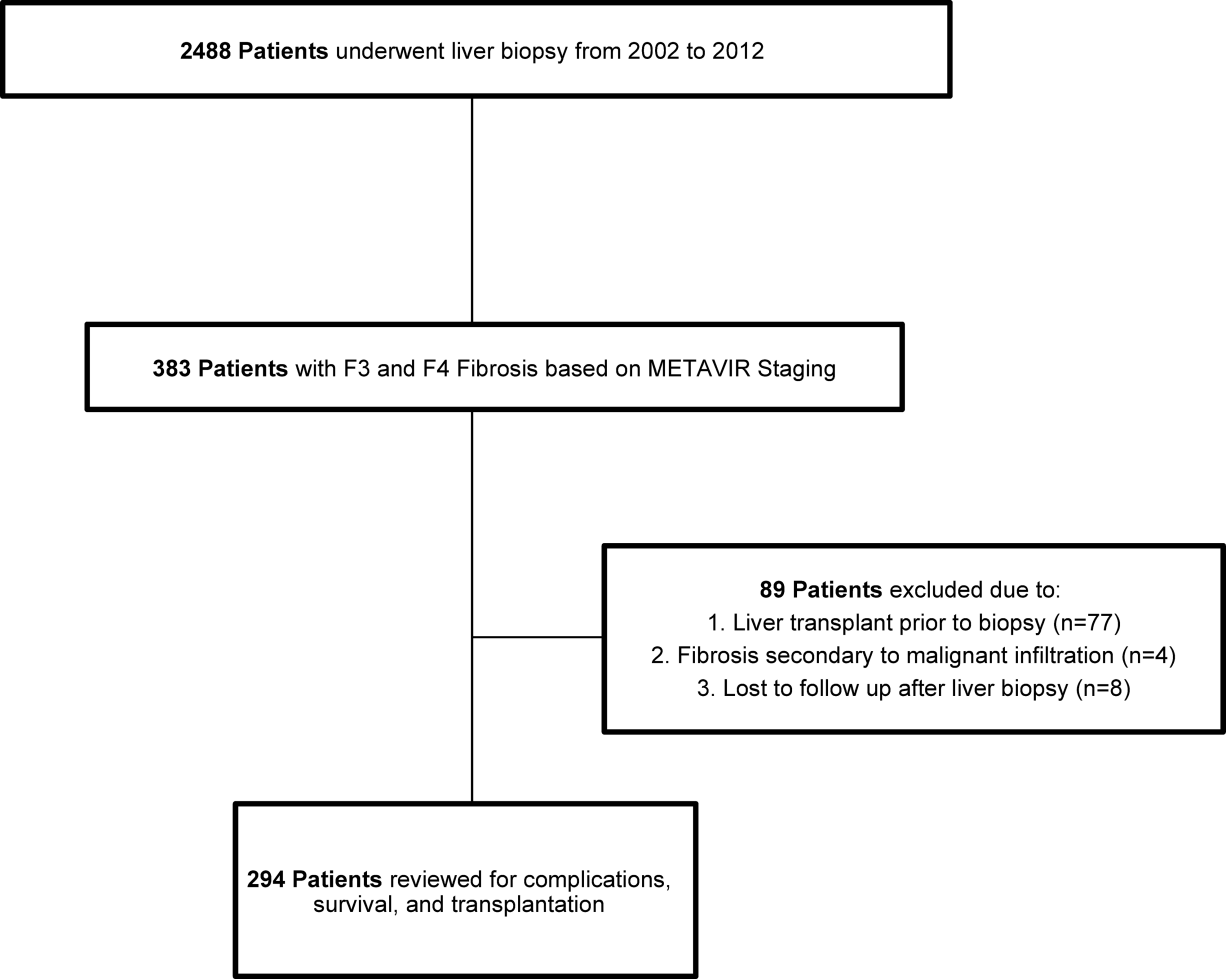
Cohort study design.

Our study is a retrospective cohort and was conducted at University of Alabama at Birmingham Hospital, a tertiary care referral center (Fig 1). We reviewed the medical charts of patients undergoing liver biopsy between January 1, 2002 and December 31, 2012. Two investigators (SM and SS) independently reviewed medical charts to select patients with F3 or F4 fibrosis on liver biopsy. In our division, all the liver biopsies are reviewed by a second pathologist during the weekly liver pathology conference for consensus on the findings including the liver fibrosis stage. Patients with prior liver transplant, fibrosis secondary to malignant infiltration, and with missing follow up data were excluded.

### Data collection

Medical charts of patients included in the study were further reviewed for information within a month from the date of liver biopsy for patient demographics (age, sex, and race); laboratory data (serum albumin, serum alanine aminotransferase [ALT], serum aspartate aminotransferase [AST], blood platelet count, serum total bilirubin, international normalized ratio [INR], and serum creatinine), etiology of liver disease, and liver disease status or decompensation. Among patients with hepatitis C virus (HCV) infection, data on HCV treatment and sustained viral response (SVR) was collected. Follow up prospective data was collected for liver disease complications among those with no decompensation at the time of liver biopsy, development of HCC, and patient survival (overall and transplant free survival). For patients without the respective outcome, data were censored at the last follow up visit. The study was carried out in accordance to the ethical guidelines of the 1975 Declaration of Helsinki and approved by the UAB Institutional Review Board for Human Use. Given the retrospective nature of our study, all patients were de-identified and individual consent was waived.

### Outcomes

Primary outcome was five-year cumulative probability of any decompensation including variceal bleeding, ascites, and/or hepatic encephalopathy, and HCC. Secondary outcomes included five-year patient survival (overall and transplant free).

### Definitions

*Fibrosis stage*: stratified on liver biopsy to stages 0 to 4 using the METAVIR scoring system: (F0—no fibrosis, F1—portal fibrosis, F2—periportal fibrosis, F3—bridging fibrosis, F4—cirrhosis). Patients with F3 and F4 were included in the study for further analysis. *Decompensation or liver disease complication*: presence of variceal hemorrhage, ascites, hepatic encephalopathy. Standard definitions for these events were used for the study. HCC was diagnosed based on accepted AASLD recommended criteria.

*Patient survival*: The survival status of each patient was confirmed with National Death Social Security Index. Time to survival was analyzed from the date of liver biopsy for both the overall and transplant free survival.

*Time to event*: For primary outcome, patients with respective outcome within thirty days of liver biopsy date were excluded for that specific analysis. For secondary outcomes on patient survival, time to event was calculated from the date of liver biopsy.

### Statistical analysis

Chi square and student’s t-tests were used for comparing the baseline characteristics at the time of liver biopsy of F3 and F4 patients for categorical and continuous variables respectively. These are presented as proportions and mean ± standard error (SE). Subgroup analysis of baseline characteristics by liver disease etiology was performed. To control for the effect of potential risk factors including liver disease etiology, age, and gender, multivariate hazard rate ratio (HR) estimates for liver disease complications were calculated by Cox proportional hazard regression analysis. Adjusted 5-year failure curves controlled for age, gender, and liver disease etiology from the stratified Cox proportional hazard model were built to show the rate of any decompensation event (variceal bleed, ascites, and hepatic encephalopathy), HCC, and for patient survival (overall and transplant free) between F3 and F4 patients. Statistical analyses were performed using SAS software (version 9.4; SAS Institute Inc, Carey, NC, USA). For all analyses, a p value of <0.05 was considered statistically significant.

## Results

### Study population

Of total of 2488 patients subjected to liver biopsy during the time period of the study, 294 patients (171 with F3) were analyzed for baseline characteristics at the time of liver biopsy (Table 1). Compared to F3, those with F4 were more likely to be males (63 vs. 43%, p = 0.001) and older (mean age 53 vs. 49 yrs., p = 0.007). As expected, serum albumin and platelets were lower in F4 compared to F3 patients (3 vs. 3.4, and 137 vs. 193, respectively), p<0.0001 for both analyzes. Alcohol use after liver biopsy was identified in 14% of patients with F3 and 12% of F4 patients (p = 0.65). A higher proportion of F4 patients were decompensated (43% vs. 16%, p<0.0001) at the time of biopsy.

**Table 1:** Baseline characteristic of patients at the time of liver biopsy.

Characteristic	F3 Fibrosis (n = 171)	F4 Fibrosis (n = 123)	*P value*
Age in yrs., mean ± SE	49 ± 0.9	53 ± 1.1	0.007
Male, n (%)	74 (43)	77 (63)	0.001
Race, n (%)			
White	130 (76)	100 (81)	0.31
African-American	35 (20)	16 (13)	
Other*	6 (4)	7 (6)	
Liver disease etiology, n (%)			
HCV	51 (30)	46 (37)	0.23
NAFLD	41 (24)	23 (19)	
ALD	20 (12)	20 (16)	
Other**	59 (34)	34 (28)	
Serum albumin (g/dl), mean ± SE	3.4 ± 0.1	3.0 ± 0.1	<0.0001
ALT (IU/l), mean ± SE	79 ± 8.3	67 ± 9.1	0.34
AST (IU/l), mean ± SE	80 ± 7.2	85 ± 7.6	0.69
Platelet count, mean ± SE	193 ± 7.8	137 ± 6.7	<0.0001
FIB-4 score, mean ± SE	3.1 ± 0.2	6.3 ± 0.9	0.0003
APRI, mean ± SE	1.4 ± 0.1	1.8 ± 0.3	0.13
Decompensated at biopsy, n (%)	28 (16)	53 (43)	<0.0001

Abbreviations: HCV: Hepatitis C virus; ALD: Alcohol-related liver disease; ALT: Alanine aminotransferase; APRI: AST to Platelet Ratio Index; AST: Aspartate aminotransferase; FIB-4: Fibrosis-4; NAFLD: Nonalcoholic liver disease; SVR: Sustained Viral Response

*Other races include Hispanic (3), Asian (n = 1), Indian (n = 2) and unknown (n = 7).

**Other etiologies include Cryptogenic (n = 27), Autoimmune Hepatitis (n = 17), Multiple etiologies (n = 16), PBC/PSC (n = 11), cardiac (n = 12), HBV (n = 4), Budd-Chiari (n = 2), Caroli Disease (n = 2), Drug-Induced (n = 1), and Sarcoidosis (n = 1)

### Comparison of baseline characteristics by liver disease

Comparing F3 and F4 patients, age was not different for all liver disease etiologies except for NAFLD (46 vs. 54 yrs., p = 0.006). F4 compared to F3 patients were more likely to be males for ALD and NAFLD liver disease etiologies (85% vs. 55% males, p = 0.04 and 35% vs. 12% males, p = 0.03, respectively). Baseline albumin and platelet counts were lower in F4 fibrosis in all liver disease etiologies (S1 Table). Across all subgroups, those with F4 fibrosis had higher rates of decompensation at time of liver biopsy. In patients with ALD, those with F4 compared to F3 were more likely to have alcohol use on follow up (60% vs. 20%, p = 0.01). Among patients with HCV related liver disease, rates were similar on receipt of HCV treatment (47 vs. 58%, p = 0.3) and on SVR (18 vs. 15%, p = 0.7).

### Five year outcomes

#### Any decompensation or liver-disease complication

**Fig 2:**
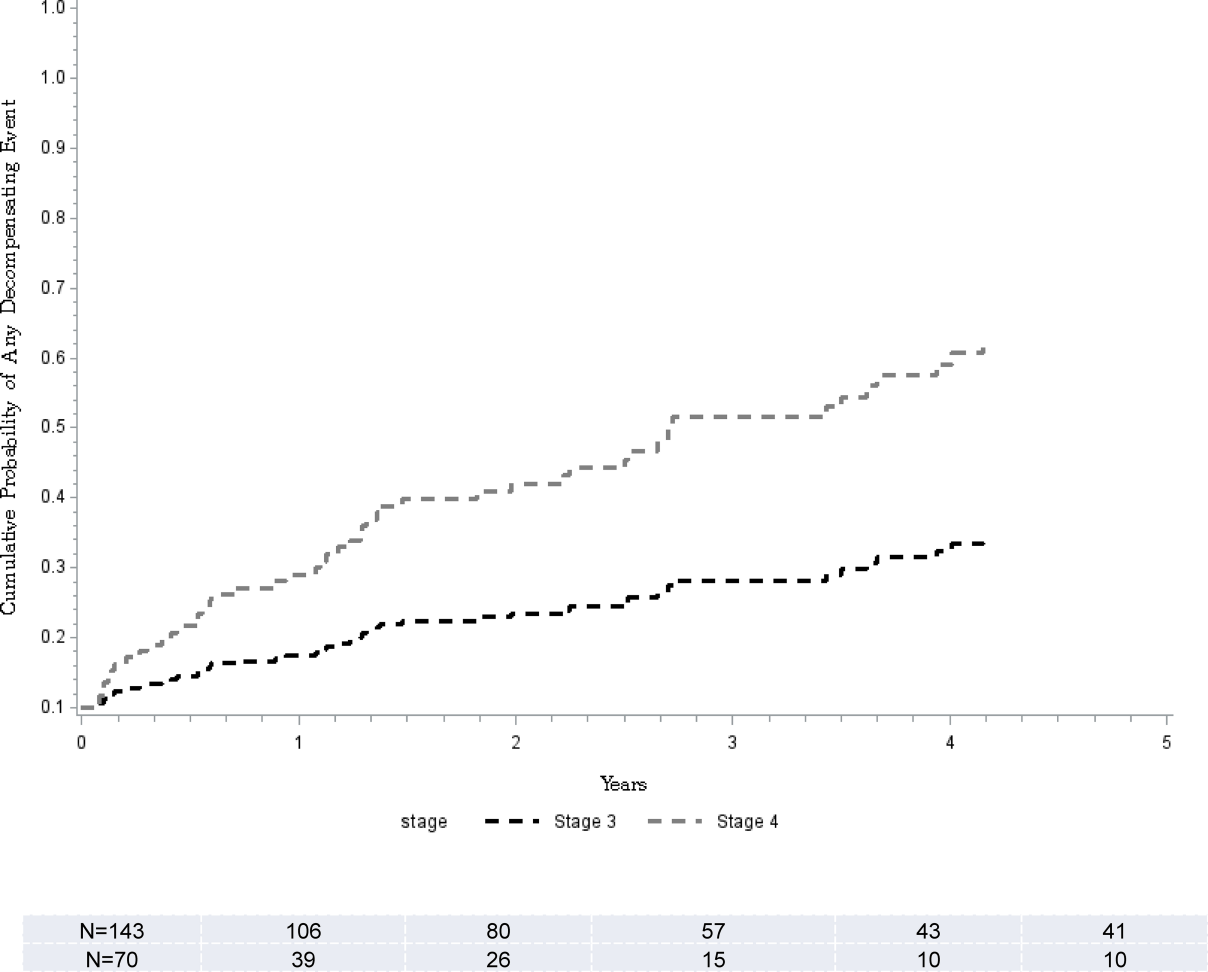
Complication-free survival. Stratified Cox model for the cumulative probability of being free of complications and time to development of any liver disease related complication comparing F3 (black line) and F4 (grey line).

Of 213 (143 F3) patients followed that had no decompensating event (variceal bleed, ascites, or hepatic encephalopathy), within the first 30 days after liver biopsy, there were a total of 58 events (25 F3) on follow up. On Cox regression model, F4 compared to F3 was associated with over 3 folds higher risk for any decompensation event (Table 2). The adjusted probability at 5 years of follow-up in patients with F4 fibrosis compared to F3 patients was higher (38% vs. 14%, p<0.0001) (Fig 2).

**Table 2:** Cox regression model for each liver disease complication.

	Hazard	95% Hazard Ratio Confidence		P Value
	Ratio	Limits		
**Any Decompensating Event**				
*Stage 4 Fibrosis*	3.28	1.89	5.68	< .0001
*Age*	1.02	0.99	1.04	0.22
*Gender*	0.53	0.31	0.92	0.02
*Liver Disease Etiology*	1.08	0.98	1.19	0.12
**Variceal Bleeding**				
*Stage 4 Fibrosis*	3.62	1.29	10.16	0.01
*Age*	0.97	0.93	1.01	0.11
*Gender*	0.88	0.32	2.40	0.80
*Liver Disease Etiology*	1.04	0.86	1.25	0.71
**Ascites**				
*Stage 4 Fibrosis*	2.54	1.21	5.35	0.01
*Age*	1.01	0.98	1.04	0.63
*Gender*	0.55	0.26	1.16	0.12
*Liver Disease Etiology*	1.02	0.88	1.18	0.80
**Hepatic Encephalopathy**				
*Stage 4 Fibrosis*	3.89	1.59	9.51	0.00
*Age*	1.01	0.98	1.05	0.47
*Gender*	1.34	0.59	3.07	0.48
*Liver Disease Etiology*	0.95	0.79	1.14	0.57
**Hepatocellular Carcinoma**				
*Stage 4 Fibrosis*	1.40	0.18	10.71	0.75
*Age*	1.00	0.92	1.09	0.96
*Gender*	0.25	0.02	2.61	0.25
*Liver Disease Etiology*	1.17	0.84	1.64	0.35
**Overall Mortality**				
*Stage 4 Fibrosis*	3.01	1.45	6.22	0.00
*Age*	1.01	0.98	1.04	0.39
*Gender*	0.86	0.44	1.69	0.66
*Liver Disease Etiology*	1.03	0.89	1.18	0.71

#### Variceal bleed

**Fig 3:**
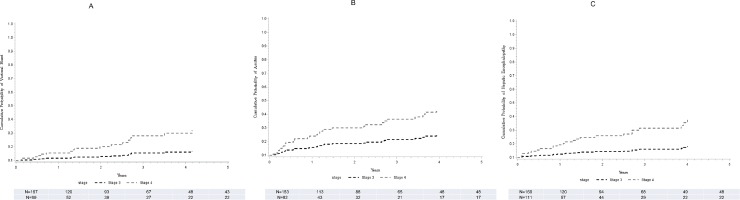
Time to development of specific liver disease complications. Stratified Cox model for the cumulative probability of being free of complications and time to development of variceal bleeding (A), ascites (B), and hepatic encephalopathy (C) comparing F3 (black line) and F4 (grey line).

Of 266 (167 F3) patients without variceal bleed at or within 30 days after liver biopsy, 17 (7 F3) developed variceal bleeding. On regression analysis, F4 compared to F3 was associated with about 3.6 folds higher risk for development of variceal bleeding (Table 2). The adjusted probability at 5 years of follow-up in patients with F4 fibrosis compared to F3 patients was higher (10 vs. 4%, p = 0.014) (Fig 3A).

#### Ascites

Of 235 (153 F3) patients without ascites at or within 30 days of liver biopsy, 31 (14 with F3) developed ascites on follow up. By Cox hazard regression analysis, patients with F4 compared to F3 stage were at over 2.5 folds higher risk for development of ascites (Table 2). There was a higher adjusted probability for ascites among F4 compared to F3 patients over 5 years follow-up (21 vs. 9%, p = 0.0014) (Fig 3B).

#### Hepatic encephalopathy

Of 280 (169 F3) patients without hepatic encephalopathy at or within 30 days of liver biopsy, 24 (8 with F3) developed hepatic encephalopathy on follow up. On Cox regression analysis model controlling for age, gender, and liver disease etiology, F4 compared to F3 patients were at about 4 folds higher risk for development of hepatic encephalopathy (Table 2). The adjusted probability at 5 years of follow-up in patients with F4 fibrosis compared to F3 patients was higher (14 vs. 5%, p = 0.003) (Fig 3C).

#### Hepatocellular carcinoma

Of 263 (165 F3) patients without HCC at or within 30 days of liver biopsy, 4 (2 with F3) developed HCC on follow up. There were no patients that developed HCC during the first 6 months follow up. On Cox regression analysis, the development of HCC was similar comparing F4 and F3 patients (Table 2). The adjusted probability at 5 years of follow-up was similar among F4 compared to F3 patients (2% vs. 1.2%, p = 0.54).

#### Overall survival

**Fig 4:**
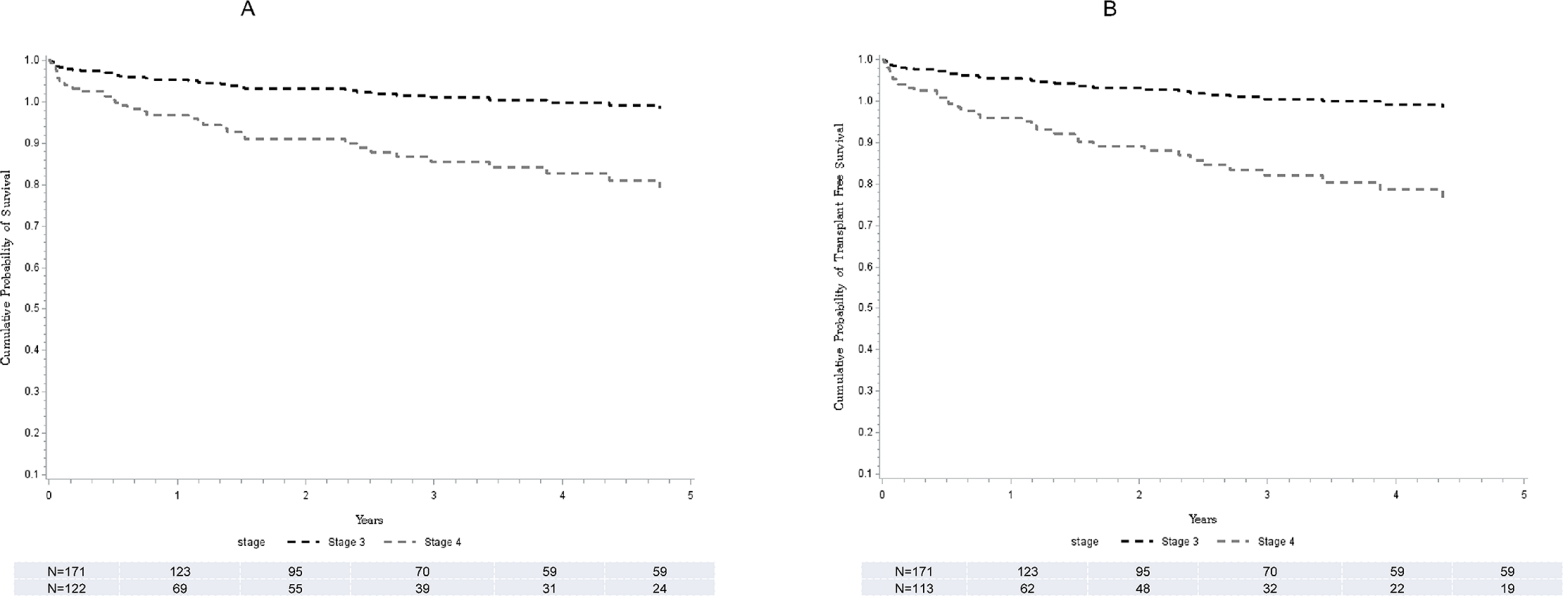
Overall survival and time to liver transplant. Stratified Cox model for the cumulative probability of survival (A) and time to orthotopic liver transplant (B) comparing F3 (black line) and F4 (grey line).

Of 293 (171 F3) patients with available survival data, 36 (12 with F3) died on follow up. The causes of death in the majority of patients were attributed to complications of advanced liver disease. By Cox hazard regression analysis, patients with F4 compared to F3 stage were at a 3 folds higher risk for death (Table 2) and there was a lower adjusted probability of overall survival at 5 years among F4 compared to F3 patients (80 vs. 93%, p = 0.003) (Fig 4A).

#### Transplant free survival

Of 284 (171 F3) patients with available survival data, 7 (all F4) patients received liver transplantation. The adjusted probability of transplant-free survival at 5 years of follow-up was lower in F4 fibrosis compared to F3 patients (80 vs. 93%, p = 0.002) (Fig 4B).

## Discussion

The main findings of our study are that patients with F4 fibrosis compared to those with F3 stage have a) higher probability of developing decompensation of liver disease including ascites, variceal bleeding, and hepatic encephalopathy and b) lower overall and transplant-free survival.

In patients with chronic HCV infection, several previous studies have reported increased rate of liver disease complications related to advanced fibrosis stage. In a study of 1050 HCV patients, 57% with Ishak stage 4 to 6, there was cumulative incidence of first liver-disease complication of 19.3% for stage 4, 37.8% for stage 5, and 49.3% for stage 6 in the 6 year follow up period.[8] The probability of liver disease complication, death, or liver transplant increased with successive fibrosis stages. Stages 4 and 5 of the Ishak system represent advanced bridging fibrosis and/or early nodule formation and have shown excellent correlation with F3 in the METAVIR system.[9]

Another study based on four large US-integrated health systems retrospectively examined 917 chronic HCV patients with F3 and F4 fibrosis for 5 years after liver biopsy.[10] For liver disease related complications comparing F4 vs. F3 fibrosis, there was increased risk for ascites (14 vs. 7.1%), esophageal varices with bleeding (4.4 vs. 1.2%), and hepatic encephalopathy (3.9 vs. 1.4%) in the 5 year observation period after liver biopsy. The 5-year survival was 77% in F4 fibrosis compared to 91% in F3 fibrosis. The 5-year probability for HCC development was 3.1% in F3 fibrosis and 8.8% in F4 fibrosis.

Huang et al. evaluated 153 patients with F3 fibrosis (mean age 45, 54% male) and F4 fibrosis (mean age 51, 54% male) over a mean follow up period of 9 years and found that F4 had significantly higher risk of liver-related complications, hepatocellular carcinoma, and death than F3 (p < 0.001).[11] Very similar to our findings, the 5-year survival in patients with F4 fibrosis was 83% compared to 96% in patients with F3 fibrosis. The 5-year probability for development of HCC patients with F3 fibrosis was 0% compared to 6% in F4 patients. At year 7, probabilities for HCC development jumped to 16% in patients with F4 fibrosis and increased to 2% in patients with F3 fibrosis. Unlike these previous 2 studies described, our study failed to show a difference in the risk for development of HCC in F3 compared to F4 fibrosis, likely because of the shorter follow up period of only 5 years and the inclusion of non-HCV patients who have lower risk for HCC development.

In NAFLD, advanced fibrosis has also been identified as leading to higher rates of liver-disease related complications and mortality.[12, 13] In a recent study of 646 patients (mean age 48, 62% male) with well-defined NAFLD followed for a mean of 20 years, patients with F4 fibrosis had a 3-fold increase in liver disease related complications and a 2-fold increase in overall mortality compared to those with F3 fibrosis.[14] The average time for patients with F3 fibrosis to develop severe liver disease as defined by the ICD-code diagnosis of cirrhosis, liver failure, HCC, or decompensated liver disease was 6 years (95% CI 2.3–9.6), however the study did not differentiate between these outcomes or provide data on HCC incidence. In another large multi-center cohort study of 619 NAFLD patients (11.5% with stage 3 or 4 fibrosis) followed for a median of 12.6 years, F4 patients compared to F3 patients had a four-fold increased probability for liver disease related complications overall and two-fold increased risk of liver-related mortality.[13] Only 3 patients in the study developed HCC, and fibrosis stage for these patients was not reported.

To our knowledge, this is the first study showing that patients with F3 compared to F4 fibrosis have lower frequency of and longer time to development of liver disease complications irrespective of liver disease etiology. Our large cohort is also well characterized with liver fibrosis stage confirmed by two separate pathologists. Further, studies have shown good inter- and intraobserver reproducibility on the fibrosis staging using any classification including the Metavir fibrosis staging system.[5] Also, potential confounders of alcohol use and HCV treatment were equally distributed in the two groups ruling out to a great extent their impact on the outcomes. However, apart from inherent limitations of a retrospective study design, our study does suffer from potential selection bias as not everyone presenting for liver disease evaluation at our center underwent a liver biopsy examination. Further, our study excluded patients with F2 fibrosis, which could be used as a control group and compare with F3 fibrosis on development of outcomes. Although for analysis on decompensation and liver disease complications, we only analyzed patients developing the respective event after 30 days from liver biopsy, it is possible that some of the F3 patients may have transitioned to F4 on follow up, and it is difficult to ascertain the stage of fibrosis at which the decompensation occurred. Repeat liver biopsy or non-invasive imaging with transient elastography was not performed and limits us from identifying the patients that may have progressed from F3 to F4 fibrosis stage.

Regardless of underlying liver disease etiology, advanced hepatic fibrosis portends increased liver-associated complications and mortality.[10–13, 15–21] However, physicians need to be vigilant as these patients unpredictably may transition to F4 stage. In this regard, data are needed on the use of non-invasive serum and radiological markers including fibroscan and transient elastography, as basis for cost-effective management of these patients in clinical practice.

In summary, our study shows lower rate of and slower development of decompensation and liver disease complications, with better overall and transplant free survival among patients with biopsy conformed bridging or advanced fibrosis (F3) as compared to patients with cirrhosis (F4) irrespective of liver disease etiology. We suggest larger multicenter prospective studies overcoming the limitations identified in this study, to confirm and validate these findings as basis for modifying guidelines and recommendations on follow up of patients with advanced fibrosis and stage 3 liver fibrosis.

## Supporting information

S1 Table Baseline patient characteristics at time of liver biopsy stratified by etiology of liver disease. (DOCX)
