# Correction: Patients with stage 3 compared to stage 4 liver fibrosis have lower frequency of and longer time to liver disease complications

**DOI:** 10.1371/journal.pone.0199402

**Published:** 2018-08-02

**Authors:** Page Axley, Sandhya Mudumbi, Shabnam Sarker, Yong-Fang Kuo, Ashwani K. Singal

The fifth author’s middle initial is missing. The correct name is: Ashwani K. Singal. The correct citation is: Axley P, Mudumbi S, Sarker S, Kuo Y-F, Singal AK (2018) Patients with stage 3 compared to stage 4 liver fibrosis have lower frequency of and longer time to liver disease complications. PLoS ONE 13(5): e0197117. https://doi.org/10.1371/journal.pone.0197117
