# Supplementary material for: Patients with stage 3 compared to stage 4 liver fibrosis have lower frequency of and longer time to liver disease complications
Source: PLoS One. 2018 May 10;13(5):e0197117. doi: 10.1371/journal.pone.0197117 (PMC5944985; doi:10.1371/journal.pone.0197117)
Supplement: S1 Table — (DOCX) [file pone.0197117.s001.docx]

**Supplementary Table 1** Baseline patient characteristics at time of liver biopsy stratified by etiology of liver disease

|  | **HCV** | | | **NAFLD** | | | **ALD** | | | **Other*** | | |
| --- | --- | --- | --- | --- | --- | --- | --- | --- | --- | --- | --- | --- |
|  | **F3**  **n=51** | **F4**  **n=46** | ***P*** | **F3**  **n=41** | **F4**  **n=23** | ***P*** | **F3**  **n=20** | **F4**  **n=20** | ***P*** | **F3**  **n=59** | **F4**  **n=34** | ***P*** |
| **Age** | 51±1.2 | 51±1.2 | 0.7 | 46±1.4 | 54±2.8 | 0.01 | 47±3.2 | 48±2.8 | 0.8 | 50±.9 | 56±2.3 | 0.6 |
| **Male** | 33(65) | 32(70) | 0.6 | 5(12) | 8(35) | 0.03 | 11(55) | 17(85) | 0.04 | 25(42) | 21(62) | 0.07 |
| **White** | 37(73) | 34(74) | 0.8 | 36(88) | 22(96) | 0.3 | 16(80) | 15(75) | 0.9 | 41(70) | 29(85) | 0.1 |
| **ALT (IU/l)** | 89±11 | 88±13 | 0.9 | 42±4.8 | 35±3.3 | 0.3 | 54±9.3 | 41±8.2 | 0.3 | 105±20 | 78±28 | 0.4 |
| **AST (IU/l)** | 85±9.9 | 100±9.9 | 0.3 | 43±5.1 | 47±4.9 | 0.6 | 75±15 | 86±15 | 0.6 | 103±17 | 90±23 | 0.6 |
| **Platelet count** | 158±8.3 | 125±8.7 | 0.01 | 223±21 | 138±20 | 0.05 | 3.5±0.2 | 2.7±0.2 | 0.01 | 3.2±0.1 | 3.1±0.1 | 0.5 |
| **Albumin (g/dl)** | 3.6±0.1 | 3.1±0.1 | 0.01 | 3.7±0.1 | 3.2±0.1 | 0.01 | 3.5±0.2 | 2.7±0.2 | 0.01 | 3.2±0.1 | 3.1±0.1 | 0.5 |
| **MELD score** | 11±0.9 | 11±0.8 | 0.7 | 9±1 | 11±1 | 0.1 | 209±23 | 172±19 | 0.02 | 207±14 | 132±11 | 0.01 |
| **Decompensated** | 9(17) | 16(35) | 0.05 | 4(10) | 7(30) | 0.04 | 13±2 | 14±1 | 0.6 | 12±0.9 | 15±1.2 | 0.06 |
| **Alcohol use** | 5(10) | 7(15) | 0.4 | 0(0) | 0(0) | 1 | 4(20) | 12(60) | 0.01 | 44(74) | 10(31) | 0.01 |

*Data expressed as number (%) or mean ± SE.*

*HCV: Hepatitis C virus; ALD: Alcoholic Liver Disease; ALT: Alanine aminotransferase; AST: Aspartate aminotransferase; MELD: Model for end-stage liver disease*

**Other etiologies include Cryptogenic, Autoimmune, Multiple etiologies, PBC/PSC, cardiac, hepatitis B, Budd-Chiari, Caroli Disease, Drug-Induced, and Sarcoidosis*
